# SARS-CoV-2 infection is associated with self-reported post-acute neuropsychological symptoms within six months of follow-up

**DOI:** 10.1371/journal.pone.0297481

**Published:** 2024-04-16

**Authors:** Liana R. Andronescu, Stephanie A. Richard, Ann I. Scher, David A. Lindholm, Katrin Mende, Anuradha Ganesan, Nikhil Huprikar, Tahaniyat Lalani, Alfred Smith, Rupal M. Mody, Milissa U. Jones, Samantha E. Bazan, Rhonda E. Colombo, Christopher J. Colombo, Evan Ewers, Derek T. Larson, Ryan C. Maves, Catherine M. Berjohn, Carlos J. Maldonado, Caroline English, Margaret Sanchez Edwards, Julia S. Rozman, Jennifer Rusiecki, Celia Byrne, Mark P. Simons, David Tribble, Timothy H. Burgess, Simon D. Pollett, Brian K. Agan

**Affiliations:** 1 Department of Preventive Medicine and Biostatistics, Infectious Disease Clinical Research Program, Uniformed Services University of the Health Sciences, Bethesda, MD, United States of America; 2 Henry M. Jackson Foundation for the Advancement of Military Medicine, Inc., Bethesda, MD, United States of America; 3 Department of Preventive Medicine and Biostatistics, Uniformed Services University of the Health Sciences, Bethesda, MD, United States of America; 4 Department of Medicine, Uniformed Services University of the Health Sciences, Bethesda, MD, United States of America; 5 Brooke Army Medical Center, San Antonio, TX, United States of America; 6 Walter Reed National Military Medical Center, Bethesda, MD, United States of America; 7 Naval Medical Center Portsmouth, Portsmouth, VA, United States of America; 8 William Beaumont Army Medical Center, El Paso, TX, United States of America; 9 Tripler Army Medical Center, Honolulu, HI, United States of America; 10 Carl R. Darnall Army Medical Center, Fort Hood, TX, United States of America; 11 Madigan Army Medical Center, Tacoma, WA, United States of America; 12 Fort Belvoir Community Hospital, Fort Belvoir, VA, United States of America; 13 Naval Medical Center San Diego, San Diego, CA, United States of America; 14 Womack Army Medical Center, Fort Bragg, NC, United States of America; Universidad de Las Americas, Quito-Ecuador, ECUADOR

## Abstract

**Background:**

Chronic neuropsychological sequelae following SARS-CoV-2 infection, including depression, anxiety, fatigue, and general cognitive difficulties, are a major public health concern. Given the potential impact of long-term neuropsychological impairment, it is important to characterize the frequency and predictors of this post-infection phenotype.

**Methods:**

The Epidemiology, Immunology, and Clinical Characteristics of Emerging Infectious Diseases with Pandemic Potential (EPICC) study is a longitudinal study assessing the impact of SARS-CoV-2 infection in U.S. Military Healthcare System (MHS) beneficiaries, i.e. those eligible for care in the MHS including active duty servicemembers, dependents, and retirees. Four broad areas of neuropsychological symptoms were assessed cross-sectionally among subjects 1–6 months post-infection/enrollment, including: depression (Patient Health Questionnaire-9), anxiety (General Anxiety Disorder-7), fatigue (PROMIS® Fatigue 7a), and cognitive function (PROMIS® Cognitive Function 8a and PROMIS® Cognitive Function abilities 8a). Multivariable Poisson regression models compared participants with and without SARS-CoV-2 infection history on these measures, adjusting for sex, ethnicity, active-duty status, age, and months post-first positive or enrollment of questionnaire completion (MPFP/E); models for fatigue and cognitive function were also adjusted for depression and anxiety scores.

**Results:**

The study population included 2383 participants who completed all five instruments within six MPFP/E, of whom 687 (28.8%) had at least one positive SARS-CoV-2 test. Compared to those who had never tested positive for SARS-CoV-2, the positive group was more likely to meet instrument-based criteria for depression (15.4% vs 10.3%, p<0.001), fatigue (20.1% vs 8.0%, p<0.001), impaired cognitive function (15.7% vs 8.6%, p<0.001), and impaired cognitive function abilities (24.3% vs 16.3%, p<0.001). In multivariable models, SARS-CoV-2 positive participants, assessed at an average of 2.7 months after infection, had increased risk of moderate to severe depression (RR: 1.44, 95% CI 1.12–1.84), fatigue (RR: 2.07, 95% CI 1.62–2.65), impaired cognitive function (RR: 1.64, 95% CI 1.27–2.11), and impaired cognitive function abilities (RR: 1.41, 95% CI 1.15–1.71); MPFP/E was not significant.

**Conclusions:**

Participants with a history of SARS-CoV-2 infection were up to twice as likely to report cognitive impairment and fatigue as the group without prior SARS-CoV-2 infection. These findings underscore the continued importance of preventing SARS-CoV-2 infection and while time since infection/enrollment was not significant through 6 months of follow-up, this highlights the need for additional research into the long-term impacts of COVID-19 to mitigate and reverse these neuropsychological outcomes.

## Introduction

As of June 2022, there were over half a billion confirmed cases of COVID-19 and more than six million deaths worldwide, with the number of actual infections exceeding this number, as variants of concern change and the pandemic continues [1,2]. Cognitive impairment and deteriorating mental health are reported by a substantial subset of patients recovering from acute COVID-19 [3–5]. However, there are conflicting reports of the direct impact of COVID-19 infection on mental health in recovering patients when compared to the general population [6].

Post-COVID conditions (PCC, often called “long COVID”) are multifaceted with symptoms that may include a combination of pain, fatigue, and cognitive dysfunction, as well as others; continuing at least 4 weeks to 3 months after contracting the virus [7,8]. At least one persistent symptom is reported in 19–72% of those infected with SARS-CoV-2, and an estimated 10% later develop PCC [9–11]. An extensive effort is underway by many researchers worldwide to better characterize the severity, duration, and risk factors of PCC. Studies have identified COVID-19 as a potential risk factor for changes in cognitive function and high rates of anxiety, depression, and fatigue, though there is a high level of heterogeneity in study design and tools used for measurement [12,13]. In EPICC (Epidemiology, Immunology, and Clinical Characteristics of Emerging Infectious Diseases with Pandemic Potential), a longitudinal study assessing the impact of SARS-CoV-2 infection, we examined the prevalence of and risk factors for depression, anxiety, fatigue, and subjective cognitive impairment in participants with and without a prior SARS-CoV-2 infection. We hypothesized that those with a history of confirmed SARS-CoV-2 infection would self-report worse cognitive-related outcomes compared to the uninfected participants, but over time these differences would diminish.

## Methods

### Study design and participant cohort

This is a cross-sectional analysis conducted as part of the EPICC prospective cohort study, which seeks to investigate the short- and long-term outcomes of SARS-CoV-2 infection among MHS beneficiaries. The EPICC study enrolled SARS-CoV-2 positive and negative participants who were eligible MHS beneficiaries and had either confirmed SARS-CoV-2 infection, COVID-like illness, a recent exposure to COVID-19, or were vaccinated against SARS-CoV-2. We used the U.S. Department of Health and Human Services definition of PCC considering persistent symptoms 4 weeks or more post-infection [8].

### Inclusion and exclusion criteria

Participants were included in our analysis if they were over 18 years of age, MHS beneficiaries, enrolled in EPICC between December 2020 and May 2022, and completed all five neuropsychological symptom questionnaires at a single timepoint by July 2022 [14]. Participants were excluded if their questionnaires were completed more than six months after the date of their first SARS-CoV-2 positive test, or enrollment for SARS-CoV-2 negative participants (MPFP/E). This study did not include data from later than six MPFP/E as a second issuance of these questionnaires will more completely address the 7–12 month timeframe and thus, our focus in this analysis was to understand early chronic outcomes (1–6 months).

### Diagnosis of SARS-CoV-2 infection, vaccination history, and demographic variables

At enrollment, participants reported demographic variables including age, sex, ethnicity, height, and weight. Any prior diagnoses of anxiety and/or depression were collected from ICD-10 codes in participants’ medical records to evaluate potential confounding as a result of a pre-existing history of depression or anxiety. A total of 687 participants were identified as SARS-CoV-2 positive based on a combination of at least one positive polymerase chain reaction (PCR) test documented in their medical record (n = 511/687 or 74.4%) and/or a self-reported PCR test (n = 668/687 or 97.2%). All others had documented or reported only negative tests and were considered SARS-CoV-2 negative. For SARS-CoV-2 positive participants, MPFP was measured as the number of months in between their first positive COVID-19 test and completion of the questionnaires. For SARS-CoV-2 negative participants, MPE was measured as the number of months in between their enrollment in the EPICC study and completion of the questionnaires. SARS-CoV-2 vaccines were made available to non-healthcare worker adult MHS beneficiaries starting April 19, 2021 [15], and vaccination status for this analysis was determined based on participants’ medical record and self-report. Disease severity for SARS-CoV-2 positive participants was categorized based on self-report as: outpatient/asymptomatic or hospitalized for COVID-19. SARS-CoV-2 positive participants reporting a first positive test date 14 days or more after the date of the final vaccination in the primary series were defined as vaccine breakthrough cases.

### Data sources and measurements

The following instruments were completed by all participants: (i) Patient Health Questionnaire-9 (PHQ-9) [16], (ii) Generalized Anxiety Disorder-7 (GAD-7) [17], (iii) PROMIS® fatigue 7a score, and (iv) PROMIS® cognitive function short form 8a and PROMIS® cognitive function short form abilities subset 8a [18]. The PHQ-9 comprises 9 multiple-choice items and the GAD-7 comprises 7 multiple-choice items. A score of 10 or higher for the PHQ-9 and GAD-7 was used to identify moderate-to-severe depression or anxiety, respectively [16,17]. PROMIS® fatigue 7a has 7 multiple-choice questions, and a t-score threshold of 60 or higher (one standard deviation above the sample mean on the PROMIS® fatigue questionnaire) identified participants with worse fatigue. The PROMIS® cognitive function short form 8a and the PROMIS® cognitive function short form abilities subset 8a each have 8 multiple-choice items. Cutoffs of less than or equal to 40 for PROMIS® cognitive function and cognitive function abilities identified participants who were one standard deviation below the sample mean (worse cognitive function). All questionnaire responses are subjective.

Both PROMIS® cognitive function questionnaires assess the participants’ perception of concentration, memory, and mental acuity, but the framing of the items is different, and it is recommended that both questionnaires be used [19]. Questionnaire invitations were sent to participants who were at least 1-month post-enrollment and repeated 6 months later to those still active in the study; invitation hyperlinks were sent via email or text message, based on the participant’s preference, and questionnaires were completed remotely via the Research Electronic Data Capture (REDCap^®^) system. To be included in the analysis, participants were required to have submitted at least one full set of questionnaires at a single timepoint. If a participant completed a full set of questionnaires on two dates, the first complete set was retained for this analysis. We assessed the correlations among the questionnaires.

### Statistical analysis

Descriptive statistics were calculated for outcome measures, demographic and physical characteristics, and comorbidities, with p-values computed using either chi-square, Fisher’s exact, or Kruskal-Wallis tests, as appropriate.

Poisson regression was performed to evaluate whether each independent variable under consideration was associated with the outcomes of interest. A full model was run which included both SARS-CoV-2 positive and negative participants to determine if history of SARS-CoV-2 infection was associated with the neuropsychological outcomes measured in EPICC, and a second model was run considering only those with a history of SARS-CoV-2 infection, to determine if certain factors were associated with poor outcomes among those exposed. Multivariable Poisson regression was performed, adjusting for covariates, which were selected *a priori*: age, sex, ethnicity, MPFP/E, active military status, body mass index (BMI), and concurrent scores for depression and anxiety [20,21]. Models assessing risk factors in the SARS-CoV-2 positive participants were also adjusted for vaccination prior to infection and disease severity.

### Ethical standards

This study was approved by the Uniformed Services University (USU) Institutional Review Board under protocol IDCRP-085; all participants provided documented informed consent.

## Results

**Fig 1:**
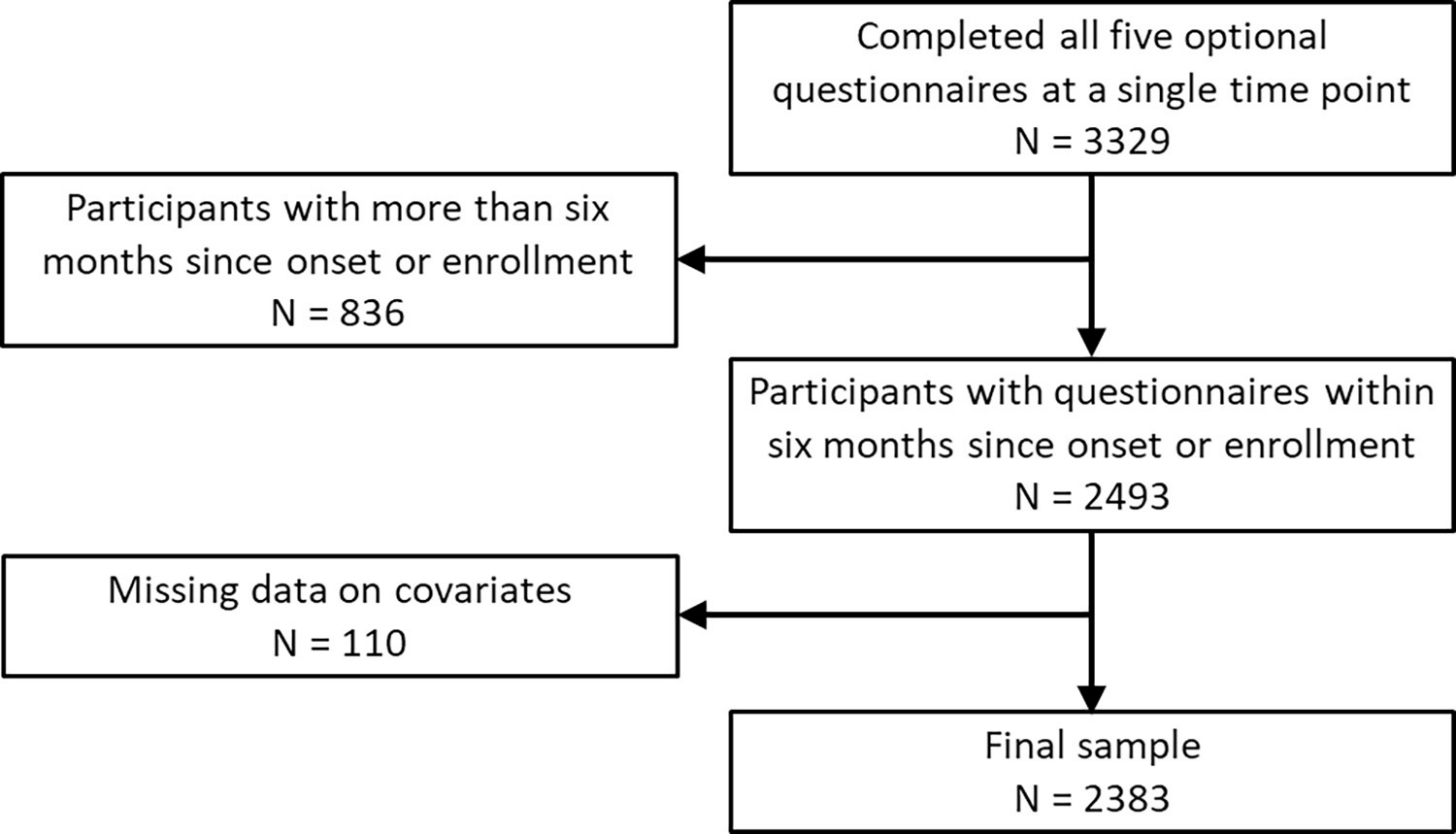
Flowchart of study population included in analysis.

There were 2383 participants with complete data and responses to all five symptom questionnaires at a single timepoint (Fig 1), 29% of whom had a history of SARS-CoV-2 infection (Table 1). A higher percentage of SARS-CoV-2 positive participants (13.0–24.3%) reported worse outcomes than SARS-CoV-2 negative participants (8.0–16.3%) across all five questionnaires, although the difference for moderate-to-severe anxiety was not statistically significant. The distribution of ethnicity did not differ significantly by SARS-CoV-2 infection status, but males and active-duty military participants were less likely to have a history of SARS-CoV-2 infection. The mean interval between enrollment and questionnaire completion for SARS-CoV-2 negative participants was 2.3 months (SD: 1.9), while SARS-CoV-2 positive participants had a mean interval between first positive test and questionnaire completion of 2.7 months (SD:1.7, p<0.001). The proportion with any prior diagnosis of depression or anxiety, as defined by ICD-10 codes, was 33.4% in the negative group and 38.4% in SARS-CoV-2 positive participants (p = 0.02).

**Table 1:** Depression, anxiety, fatigue, self-assessed cognitive function, and general characteristics of EPICC study participants, by SARS-CoV-2 infection status.

	Total (N = 2383)	SARS-CoV-2 negative (N = 1696)	SARS-CoV-2 positive (N = 687)	P-value^1^
**Outcomes, n (%)**				
Depression (PHQ-9 score ≥ 10)	281 (11.8%)	175 (10.3%)	106 (15.4%)	< 0.001
Anxiety (GAD-7 score ≥ 10)	279 (11.7%)	190 (11.2%)	89 (13.0%)	0.228
Fatigue (PROMIS® 7a t-score ≥ 60)	274 (11.5%)	136 (8.0%)	138 (20.1%)	< 0.001
Impaired cognitive function (PROMIS® 8a t-score ≤ 40)	254 (10.7%)	146 (8.6%)	108 (15.7%)	< 0.001
Impaired cognitive function abilities (PROMIS® subset 8a t-score ≤ 36)	444 (18.6%)	277 (16.3%)	167 (24.3%)	< 0.001
**Male, n (%)**	1558 (65.4%)	1140 (67.2%)	418 (60.8%)	0.003
**Ethnicity, n (%)**				0.499
White	1564 (65.6%)	1120 (66.0%)	444 (64.6%)	
Black	159 (6.7%)	111 (6.5%)	48 (7.0%)	
Hispanic or Latino	318 (13.3%)	216 (12.7%)	102 (14.8%)	
Other	342 (14.4%)	249 (14.7%)	93 (13.5%)	
**Active military duty, n (%)**	1958 (82.2%)	1440 (84.9%)	518 (75.4%)	< 0.001
**Age, mean (SD)**	37.5 (10.3)	37.3 (10.2)	37.9 (10.6)	0.384
**BMI, mean (SD)**	27.7 (4.6)	27.4 (4.4)	28.2 (5.0)	<0.001
**Months post first positive or enrollment, mean (SD)**	2.4 (1.9)	2.3 (1.9)	2.7 (1.7)	< 0.001
**Prior diagnosis of depression and/or anxiety, n (%)**	831 (34.9%)	567 (33.4%)	264 (38.4%)	0.020
**Vaccine breakthrough, n (%)**	--	--	476 (69.3%)	
**COVID-19 severity, n (%)**				
Outpatient/Asymptomatic	--	--	648 (94.3%)	
Hospitalized	--	--	39 (5.7%)	

^1^Comparing SARS-CoV-2 negative and positive groups using chi-square or Kruskal-Wallis tests.

### Association between SARS-CoV-2 history and risk of impaired mental health and cognition

**Fig 2:**
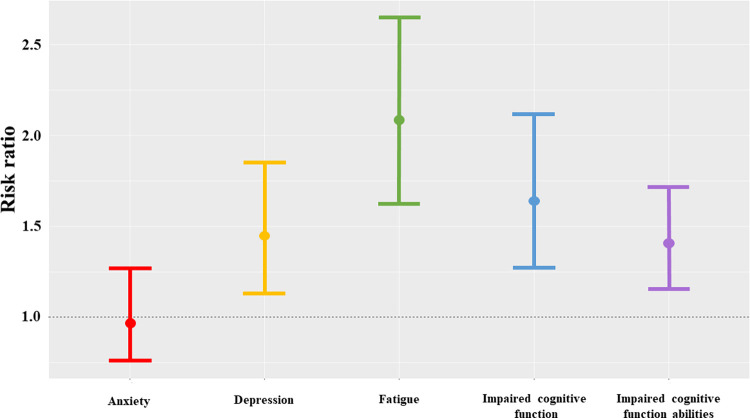
Depression, fatigue and self-assessed cognitive impairment^1^ are more prevalent in SARS-CoV-2 positive participants compared to negative participants^2^ participating in the EPICC study. ^1^ Poor outcome defined as lowest 10% of scores for anxiety and depression, and 1 standard deviation below the population mean for fatigue, impaired cognitive function, and impaired cognitive function abilities. ^2^ Multivariable Poisson regression adjusting for time since first positive test (or time since enrollment for SARS-CoV-2 negative participants), sex, ethnicity, age, BMI, and active-duty military. Models measuring fatigue, cognitive functions, and cognitive abilities were also adjusted for the depression and anxiety scores.

Participants positive for SARS-CoV-2 had increased risks of moderate-to-severe depression (RR: 1.44, 95% CI 1.12–1.84), fatigue (RR: 2.07, 95% CI 1.62–2.65), impaired cognitive function (RR: 1.64, 95% CI 1.27–2.11), and impaired cognitive function abilities (RR: 1.41, 95% CI 1.15–1.71) after adjusting for MPFP/E, sex, ethnicity, military active-duty status, age, and BMI (Fig 2). SARS-CoV-2 infection was not statistically significantly associated with moderate-to-severe anxiety (RR: 0.98, 95% CI: 0.75–1.26). Detailed model results are available in S1 Table. To reduce the risk of model over-fitting, prior histories of anxiety or depression were not retained in the final model, however sensitivity analyses excluding those with prior diagnoses showed similar associations.

### Risk factors among SARS-CoV-2 positive individuals

Among participants with a history of SARS-CoV-2 infection, depression was associated with increased risk of anxiety (RR: 1.19, 95% CI: 1.15–1.23), fatigue (RR: 1.18, 95% CI: 1.14–1.23), impaired cognitive function (RR: 1.14, 95% CI: 1.09–1.19), and impaired cognitive function abilities (RR: 1.09, 95% CI: 1.05–1.13) (Table 2). Anxiety was also associated with increased risk of depression in this population (RR: 1.18, 95% CI: 1.14–1.21). Active-duty military participants were at increased risk of fatigue (RR: 1.70, 95% CI: 1.00–2.99) and impaired cognitive function (RR: 2.33, 95% CI: 1.24–4.62). Ethnicity, age, BMI, MPFP/E, prior vaccination, and disease severity were not significantly associated with increased risk of moderate-to-severe depression, anxiety, or impaired cognitive function and abilities.

**Table 2:** Poisson regression of potential risk factors for moderate-to-severe depression and anxiety, increased fatigue, and self-assessed cognitive impairment among SARS-CoV-2 positive participants (N = 687)- aRR (95% CI)^1^^,^^2^^,^^3^.

	PHQ-9^4^ Depression	GAD-7^5^ Anxiety	PROMIS® 8a^6^ Fatigue	PROMIS® short form 4a^7^ Impaired Cognitive Function	PROMIS® short form subset 4a^8^ Impaired Cognitive Function Abilities
**Months post first positive test**	1.04 (0.92–1.17)	0.95 (0.83–1.07)	0.94 (0.84–1.04)	1.05 (0.94–1.18)	1.03 (0.94–1.13)
**Male**	1.00 (0.65–1.54)	0.70 (0.44–1.12)	0.69 (0.48–1.00)	0.82 (0.54–1.25)	0.86 (0.62–1.21)
**Race/ethnicity**					
White	REF	REF	REF	REF	REF
Black	1.97 (1.00–3.59)	0.95 (0.41–1.92)	1.49 (0.84–2.47)	0.53 (0.18–1.19)	1.11 (0.6–1.89)
Hispanic or Latino	1.55 (0.93–2.50)	0.69 (0.36–1.25)	1.03 (0.64–1.62)	1.02 (0.60–1.66)	1.23 (0.81–1.82)
Other	0.63 (0.26–1.30)	1.05 (0.50–1.99)	1.19 (0.68–1.98)	0.76 (0.36–1.43)	0.76 (0.42–1.27)
**Active-duty military**	0.95 (0.55–1.69)	1.12 (0.59–2.16)	**1.70 (1.00–2.99)**	**2.33 (1.24–4.62)**	1.56 (0.97–2.56)
**Age**	1.00 (0.98–1.02)	1.01 (0.99–1.03)	1.01 (0.99–1.03)	1.02 (1–1.04)	1.02 (1.00–1.03)
**BMI**	1.02 (0.98–1.06)	1.00 (0.96–1.04)	1.02 (0.98–1.05)	1.01 (0.97–1.05)	1.01 (0.97–1.04)
**Disease severity**					
Outpatient	REF	REF	REF	REF	REF
Hospitalized	1.36 (0.67–2.6)	0.62 (0.21–1.49)	1.59 (0.85–2.8)	1.34 (0.61–2.7)	0.86 (0.43–1.56)
**Vaccine breakthrough**	1.16 (0.74–1.86)	0.69 (0.42–1.13)	0.95 (0.64–1.44)	1.14 (0.72–1.84)	0.81 (0.57–1.16)
**Depression (PHQ-9 continuous scores)**		**1.19 (1.15–1.23)**	**1.18 (1.14–1.23)**	**1.14 (1.09–1.19)**	**1.09 (1.05–1.13)**
**Anxiety (GAD-7 continuous scores)**	**1.18 (1.14–1.21)**		0.97 (0.93–1.01)	1.03 (0.98–1.07)	1.04 (1.00–1.08)

^1^ aRR- adjusted rate ratio; CI- confidence interval.

^2^ All models adjusted for time since first positive test or enrollment, sex, ethnicity, active duty military, age, BMI, disease severity, vaccine breakthrough, and concurrent depression and anxiety scores from PHQ-9 and GAD-7 questionnaires.

^3^ Statistically significant aRR at p<0.05 are in bold text.

^4^ PHQ-9 has a cutoff score of ≥10 to identify participants with moderate-to-severe depression.

^5^ GAD-7 has a cutoff score of ≥10 to identify participants with moderate-to-severe anxiety.

^6^ PROMIS® 8a Fatigue questionnaire has a cutoff of ≥60 to identify participants one standard deviation above the sample mean.

^7^ PROMIS® cognitive function short form 4a has a cutoff of ≤40 to identify participants one standard deviation below the sample mean.

^8^ PROMIS® cognitive function short form abilities subset 4a has a cutoff of ≤36 to identify participants one standard deviation below the sample mean.

## Discussion

In this prospective cohort study, using data from the initial neuropsychological follow-up questionnaires, we found a significantly higher proportion of participants with a history of SARS-CoV-2 infection reporting moderate-to-severe depression and anxiety, increased fatigue, and more pronounced cognitive impairment up to 6 months post infection compared to those without SARS-CoV-2 infection. Although responses to each questionnaire were highly correlated, the increased risk for depression, fatigue, and cognitive impairment remained after controlling for depression and anxiety, as well as other demographic characteristics. Additionally, the results of our analyses for cognitive symptoms were not altered by time-since-enrollment or first positive test, whether comparing all participants by SARS-CoV-2 infection status or assessing risk factors for poor outcomes among SARS-CoV-2 infected participants alone. In addition, anxiety, depression, and fatigue symptoms were not associated with MPSO/E among SARS-CoV-2 positive participants. Further investigation of the presence and persistence of neuropsychological outcomes of SARS-CoV-2 infection with repeated measures at the subject level through one year of follow-up is needed and ongoing.

The prevalence of fatigue and cognitive impairment among participants with a history of SARS-CoV-2 infection is consistent with the available body of literature for recovering COVID-19 patients [22], but our study was able to determine the increased risk for our measured outcomes compared with study participants without a history of SARS-CoV-2 infection. Consistent with our findings, persistent fatigue and self-reported cognitive impairment following COVID-19 illness has been reported in other studies [4,11,22,23]. One systematic review reported that up to 32% of recovering patients reported fatigue and 22% reported cognitive impairment after at least 12 weeks of follow-up [3]. That proportion of fatigue is approximately double the amount identified in our own recovering patients (14.9%) and the proportion of cognitive impairment is close to our finding of 14.2–24.5% among recovering patients.

Overall, participants in the EPICC cohort reported a high prevalence of anxiety and depression which is consistent with other studies, including those using the same PHQ-9 and GAD-7 instruments [24,25]. Our study assessed the correlations among the questionnaires used and found a high degree of correlation. PROMIS® cognition scores have previously been shown to correlate with PHQ-9 and GAD-7, with approximately 42% of depressed and anxious patients reporting problems with memory and concentration in a non-COVID-19 population [26]. Even after adjusting for PHQ-9 and GAD-7 scores, the association of worse cognitive symptoms with SARS-CoV-2 infection remained, reinforcing the impact of COVID-19 on cognition. While anxiety as a variable did not reach statistical significance in any associations with SARS-CoV-2 infection, this could be confounded by social concerns unique to the phases of the pandemic’s evolution as well as high rates of general anxiety within the overall population that could exert an effect that functions differently than other mental health conditions.

Our analysis indicates an increased risk for depression, fatigue, and cognitive impairment symptoms among patients recovering from COVID-19, though their long-term persistence or resolution is not yet known. Other longitudinal studies also found that various post-infectious symptoms of COVID-19 syndrome may variably persist or worsen for at least a year following illness [27–29]. Symptoms such as fatigue and cognitive impairment disrupt both the work and social life of recovering patients, which has significant implications for quality of life and may even contribute to ongoing symptoms of depression and anxiety [30–32]. As longitudinal data continue to be collected, it is imperative to assess how long symptoms persist and what might be done to reduce the symptom burden on patients.

Among participants with COVID-19, comorbid depression was identified as a risk factor for anxiety, fatigue, and self-assessed cognitive impairment, while anxiety was a risk factor for depression and impaired cognitive function abilities. Active-duty military status was also associated with increased risk of fatigue and impaired cognitive function. These findings are striking as they may indicate that self-reported post-COVID-19 cognitive impairment is difficult to predict based on patient characteristics at the time of initial SARS-CoV-2 infection. Moreover, the lack of association between either vaccination or initial COVID-19 severity and post-COVID-19 self-reported cognitive impairment may imply that this complication remains challenging to mitigate among those who acquire SARS-CoV-2 infection. However, our study had limited power to detect a difference in risk based on disease severity because very few participants were hospitalized, though previous studies have identified hospitalization as a predictor of depression and anxiety [24]. Because other studies reported associations of age and sex with persistent symptoms after COVID-19, we included those variables in our models *a priori*. However, they were not independently associated with risk for depression, anxiety, fatigue, or cognitive impairment in our models [33,34]. Previous studies have documented an association between ethnicity, distress, depression, and anxiety including related to COVID-19 [35–39] although some have shown an absence of association with persistent neurocognitive symptoms, e.g. [40]; ethnicity was not associated with risk for depression, anxiety, fatigue, or cognitive impairment among participants with a history of SARS-CoV-2 infection in this study. Although we did not identify any demographic risk factors, there are other potential risk factors for PCC that were not measured in our study, such as genetic factors, which merit further study [41,42].

The absence of an association in our study between ethnicity and the symptom groups studied is noteworthy. The EPICC study has previously shown race and ethnicity is associated with COVID-19 hospitalization risk, but race and ethnicity was not associated with persistent symptoms (of any kind) in another EPICC analysis [43,44]. As noted above, a number of studies have shown increased distress, mental health, or cognitive symptoms among non-white COVID-19 populations and efforts to understand these disparities have demonstrated important underlying differences in socioeconomic status and economic impact [37,45], discrimination and racial bias [37], and family activity disruption [45]. An NHANES-based study of depression and race/ethnicity identified differences in financial, physical, and social assets as underlying factors [35]; after adjusting for these, non-Hispanic Black and Hispanic persons, who had higher odds in unadjusted analyses, had 0.8 times lower odds of probable depression than non-Hispanic Whites, consistent with reports suggesting increased resilience among non-White groups despite other disparities [46,47]. Taken together these studies demonstrate the importance of addressing underlying disparities related to health outcomes including for persistent COVID-19 related neurocognitive symptoms.

While earlier studies primarily focused on persistent symptoms in COVID-19 patients with severe initial presentations, our SARS-CoV-2 positive participants were less severely ill, with presentations ranging mostly from asymptomatic to moderately ill [48,49]. Despite small numbers of hospitalized patients in our study, illness severity did not impact risk of cognitive impairment and poor outcomes, which occurred at all levels of COVID-19 severity. While this phenomenon may not be generalizable to the hospitalized or critically ill population, it is congruent with findings of other studies that specifically assessed patients recovering from mild to moderate episodes of COVID-19 [21,50–52]. Therefore, even as the presentation of COVID-19 diminishes with the evolution of the pandemic in an immunologically non-naïve population, the post-infectious morbidity burden of cognitive and psychiatric impacts may persist.

The existing body of research on depression, anxiety, fatigue, and cognitive impairment following COVID-19 diagnosis uses a wide variety of measures such as subjective self-reporting via online questionnaires or clinical follow-up, evaluation by physicians, phone interviews, and instruments such as RAND-36, SPHERE-34, FACIT, MoCA, and the Barthel index [3]. This variety of measures makes it difficult to directly compare results of one self-assessment to another. Systematic reviews have identified the variety of study designs as one of the barriers to drawing conclusions on studies assessing persistent COVID-19 symptoms [10]. By utilizing widely available instruments such as PHQ-9 and the PROMIS® questionnaires our results are comparable to the studies that also made use of these tools [11,24].

This study was limited by potential selection bias of those who responded to the questionnaires and includes primarily SARS-CoV-2 positive participants with minimal acute disease severity. Since the questionnaires had 32 total questions when counted together, participants may have become fatigued while answering introducing potential bias. However, all participants completed the same set of questionnaires, regardless of exposure status, and any fatigue from completing the questionnaires would likely be evenly distributed between those with or without SARS-CoV-2 infection. We were also limited by the absence of pre-enrollment or pre-pandemic baseline measures of cognition and fatigue, and therefore, it is unknown how many participants may have had similar symptoms prior to enrollment or COVID-19 diagnosis, potentially exaggerating the findings. However, with the adjustments for concurrent PHQ-9 and GAD-7 scores, we were able to limit this effect and demonstrate that there is an independently increased risk of perceived cognitive impairment and fatigue controlling for ongoing depression and anxiety symptoms in the setting of SARS-CoV-2 infection. We also compared participants with a prior diagnosis of anxiety or depression with those without and found that they did not differ by age or interval since enrollment or first positive test (S2 Table). Within the participants without a history of mental illness, we still saw significantly increased risk of symptoms of depression, fatigue, and impaired cognitive function. The EPICC study is ongoing, and participants continue to complete the questionnaires at regular intervals, allowing for continued longitudinal analysis of trends in recovery or ongoing symptoms of depression, anxiety, fatigue, and cognitive impairment. Further assessment of cognitive performance using neuropsychological tests is also planned.

## Conclusions

Ongoing moderate-to-severe depression, fatigue, and cognitive impairment were reported by a statistically significantly higher proportion of SARS-CoV-2 positive patients compared to those without evidence of SARS-CoV-2 infection. Notably, fatigue seemed to attenuate in accordance with longer intervals between first positive test and questionnaire completion, which may provide a source of optimism for the potential reversibility of other symptoms over a longer period. However, given the high prevalence of reported depression, anxiety, fatigue, and cognitive impairment within six months from study enrollment or first positive test, further characterization is recommended to determine how long these symptoms persist and whether other risk factors may be associated with prolonged symptoms. The prevention of SARS-CoV-2 infection is important to avoid associated morbidity. Evaluation of treatment strategies to ameliorate PCC, including neuropsychological symptoms, remains a priority given the continuing large number of cases; clinical trials and analyses of cognitive behavioral therapy, antidepressants and other medications, exercise, and additional novel approaches are ongoing and may offer hope to patients with persistent neuropsychological symptoms.

### Disclaimer

The contents of this publication are the sole responsibility of the author (s) and do not necessarily reflect the views, opinions, or policies of Uniformed Services University of the Health Sciences (USUHS); the Department of Defense (DoD); the Departments of the Army, Navy, or Air Force; the Defense Health Agency, Brooke Army Medical Center; Walter Reed National Military Medical Center; Naval Medical Center San Diego; Madigan Army Medical Center; United States Air Force School of Aerospace Medicine; Fort Belvoir Community Hospital; Carl R. Darnall Army Medical Center; Naval Medical Center Portsmouth; Tripler Army Medical Center; United States Coast Guard; Womack Army Medical Center; William Beaumont Army Medical Center; US Army Medical Department; US Army Office of the Surgeon General; the Henry M. Jackson Foundation for the Advancement of Military Medicine, Inc; the National Institutes of Health. Mention of trade names, commercial products, or organizations does not imply endorsement by the U.S. Government. The investigators have adhered to the policies for protection of human subjects as prescribed in 45 CFR 46.

## Supporting information

S1 Table Poisson regression measuring the risk of moderate-to-severe depression and anxiety, increased fatigue, and self-assessed cognitive impairment by COVID-19 infection (N = 2383)- aRR (95% CI). (PDF)

S2 Table Depression, anxiety, fatigue, self-assessed cognitive function, and demographics of EPICC study participants by history of anxiety and/or depression diagnosis. (PDF)
