# Correction: SARS-CoV-2 infection is associated with self-reported post-acute neuropsychological symptoms within six months of follow-up

**DOI:** 10.1371/journal.pone.0351062

**Published:** 2026-06-03

**Authors:** Liana R. Andronescu, Stephanie A. Richard, Ann I. Scher, David A. Lindholm, Katrin Mende, Anuradha Ganesan, Nikhil Huprikar, Tahaniyat Lalani, Alfred Smith, Rupal M. Mody, Milissa U. Jones, Samantha E. Bazan, Rhonda E. Colombo, Christopher J. Colombo, Evan Ewers, Derek T. Larson, Ryan C. Maves, Catherine M. Berjohn, Carlos J. Maldonado, Caroline English, Margaret Sanchez Edwards, Julia S. Rozman, Jennifer Rusiecki, Celia Byrne, Mark P. Simons, David Tribble, Timothy H. Burgess, Simon D. Pollett, Brian K. Agan

Tables 1, 2, S1, and S2 Tables were uploaded incorrectly. Please see the correct versions below.

**Table 1:** Depression, anxiety, fatigue, self-assessed cognitive function, and general characteristics of EPICC study participants, by SARS-CoV-2 infection status.

	Total (N = 2383)	SARS-CoV-2 negative (N = 1696)	SARS-CoV-2 positive (N = 687)	P-value^1^
**Outcomes, n (%)**				
Depression (PHQ-9 score ≥ 10)	281 (11.8%)	175 (10.3%)	106 (15.4%)	< 0.001
Anxiety (GAD-7 score ≥ 10)	279 (11.7%)	190 (11.2%)	89 (13.0%)	0.228
Fatigue (PROMIS® 7a t-score ≥ 60)	274 (11.5%)	136 (8.0%)	138 (20.1%)	< 0.001
Impaired cognitive function (PROMIS® 8a t-score ≤ 40)	254 (10.7%)	146 (8.6%)	108 (15.7%)	< 0.001
Impaired cognitive function abilities (PROMIS® subset 8a t-score ≤ 40)	444 (18.6%)	277 (16.3%)	167 (24.3%)	< 0.001
**Male, n (%)**	1558 (65.4%)	1140 (67.2%)	418 (60.8%)	0.003
**Ethnicity, n (%)**				0.499
White	1564 (65.6%)	1120 (66.0%)	444 (64.6%)	
Black	159 (6.7%)	111 (6.5%)	48 (7.0%)	
Hispanic or Latino	318 (13.3%)	216 (12.7%)	102 (14.8%)	
Other	342 (14.4%)	249 (14.7%)	93 (13.5%)	
**Active military duty, n (%)**	1958 (82.2%)	1440 (84.9%)	518 (75.4%)	< 0.001
**Age, mean (SD)**	37.5 (10.3)	37.3 (10.2)	37.9 (10.6)	0.384
**BMI, mean (SD)**	27.7 (4.6)	27.4 (4.4)	28.2 (5.0)	<0.001
**Months post symptom onset or enrollment, mean (SD)**	2.4 (1.9)	2.3 (1.9)	2.7 (1.7)	< 0.001
**Prior diagnosis of depression and/or anxiety, n (%)**	831 (34.9%)	567 (33.4%)	264 (38.4%)	0.020
**Vaccine breakthrough, n (%)**	--	--	476 (69.3%)	
**COVID-19 severity, n (%)**				
Outpatient/Asymptomatic	--	--	648 (94.3%)	
Hospitalized	--	--	39 (5.7%)	

^1^Comparing SARS-CoV-2 negative and positive groups using chi-square or Kruskal-Wallis tests.

**Table 2:** Poisson regression of potential risk factors for moderate-to-severe depression and anxiety, increased fatigue, and self-assessed cognitive impairment among SARS-CoV-2 positive participants (N = 687)- aRR (95% CI)^1^^,^^2^^,^^3^.

	PHQ-9^4^ Depression	GAD-7^5^ Anxiety	PROMIS® 7a^6^ Fatigue	PROMIS® short form 8a^7^ Impaired Cognitive Function	PROMIS® short form 8a^^8^^ Impaired Cognitive Function Abilities
**Months post first positive test**	1.04 (0.92-1.17)	0.95 (0.83-1.07)	0.94 (0.84-1.04)	1.05 (0.94-1.18)	1.03 (0.94-1.13)
**Male**	1.00 (0.65-1.54)	0.70 (0.44-1.12)	0.69 (0.48-1.00)	0.82 (0.54-1.25)	0.86 (0.62-1.21)
**Race/ethnicity**					
White	REF	REF	REF	REF	REF
Black	1.97 (1.00-3.59)	0.95 (0.41-1.92)	1.49 (0.84-2.47)	0.53 (0.18-1.19)	1.11 (0.6-1.89)
Hispanic or Latino	1.55 (0.93-2.50)	0.69 (0.36-1.25)	1.03 (0.64-1.62)	1.02 (0.60-1.66)	1.23 (0.81-1.82)
Other	0.63 (0.26-1.30)	1.05 (0.50-1.99)	1.19 (0.68-1.98)	0.76 (0.36-1.43)	0.76 (0.42-1.27)
**Active-duty military**	0.95 (0.55-1.69)	1.12 (0.59-2.16)	**1.70 (1.00-2.99)**	**2.33 (1.24-4.62)**	1.56 (0.97-2.56)
**Age**	1.00 (0.98-1.02)	1.01 (0.99-1.03)	1.01 (0.99-1.03)	1.02 (1-1.04)	1.02 (1.00-1.03)
**BMI**	1.02 (0.98-1.06)	1.00 (0.96-1.04)	1.02 (0.98-1.05)	1.01 (0.97-1.05)	1.01 (0.97-1.04)
**Disease severity**					
Outpatient	REF	REF	REF	REF	REF
Hospitalized	1.36 (0.67-2.6)	0.62 (0.21-1.49)	1.59 (0.85-2.8)	1.34 (0.61-2.7)	0.86 (0.43-1.56)
**Vaccine breakthrough**	1.16 (0.74-1.86)	0.69 (0.42-1.13)	0.95 (0.64-1.44)	1.14 (0.72-1.84)	0.81 (0.57-1.16)
**Depression (PHQ-9 continuous scores)**		**1.19 (1.15-1.23)**	**1.18 (1.14-1.23)**	**1.14 (1.09-1.19)**	**1.09 (1.05-1.13)**
**Anxiety (GAD-7 continuous scores)**	**1.18 (1.14-1.21)**		0.97 (0.93-1.01)	1.03 (0.98-1.07)	1.04 (1.00-1.08)

^1^ aRR- adjusted rate ratio; CI- confidence interval.

^2^ All models adjusted for time since first positive test or enrollment, sex, ethnicity, active duty military, age, BMI, disease severity, vaccine breakthrough, and concurrent depression and anxiety scores from PHQ-9 and GAD-7 questionnaires.

^3^ Statistically significant aRR at p < 0.05 are in bold text.

^4^ PHQ-9 has a cutoff score of ≥10 to identify participants with moderate-to-severe depression.

^5^ GAD-7 has a cutoff score of ≥10 to identify participants with moderate-to-severe anxiety.

^6^ PROMIS® 7a Fatigue questionnaire has a cutoff of ≥60 to identify participants one standard deviation above the sample mean.

^7^ PROMIS® cognitive function short form 8a has a cutoff of ≤40 to identify participants one standard deviation below the sample mean.

^8^ PROMIS® cognitive function short form abilities 8a has a cutoff of ≤40 to identify participants one standard deviation below the sample mean.

## Supporting information

S1 Table Poisson regression measuring the risk of moderate-to-severe depression and anxiety, increased fatigue, and self-assessed cognitive impairment by COVID-19 infection (N = 2383)- aRR (95% CI). (PDF)

S2 Table Depression, anxiety, fatigue, self-assessed cognitive function, and demographics of EPICC study participants by history of anxiety and/or depression diagnosis. (PDF)
