# Supplementary material for: SARS-CoV-2 infection is associated with self-reported post-acute neuropsychological symptoms within six months of follow-up
Source: PLoS One. 2024 Apr 16;19(4):e0297481. doi: 10.1371/journal.pone.0297481 (PMC11020833; doi:10.1371/journal.pone.0297481)
Supplement: S1 Table — (PDF) [file pone.0297481.s001.pdf]

S1 Table. Poisson regression measuring the risk of moderate-to-severe depression and anxiety, increased fatigue, and self-assessed cognitive impairment by COVID-19 infection (N=2383)- aRR (95% CI)[1,2,3]

	PHQ-9[2] Depression	GAD-7[3] Anxiety	PROMIS® 8a[4] Fatigue	PROMIS® short form 4a[5] Impaired Cognitive Function	PROMIS® short form subset 4a[6] Impaired Cognitive Function Abilities
**SARS-CoV-2 positive**	**1.44 (1.12-1.84)**	0.98 (0.75-1.26)	**2.07 (1.62-2.65)**	**1.64 (1.27-2.11)**	**1.41 (1.15-1.71)**
**Months post symptom onset**	0.96 (0.90-1.02)	1.01 (0.95-1.08)	0.97 (0.91-1.04)	0.99 (0.92-1.05)	0.99 (0.94-1.04)
**Male**	0.90 (0.70-1.16)	0.77 (0.60-1.00)	0.78 (0.60-1.01)	0.87 (0.66-1.14)	0.90 (0.74-1.11)
**Race/ethnicity**					
White	REF	REF	REF	REF	REF
Black	**1.75 (1.17-2.54)**	0.88 (0.57-1.31)	1.15 (0.77-1.67)	0.73 (0.44-1.15)	1.26 (0.89-1.73)
Hispanic or Latino	**1.41 (1.02-1.91)**	0.83 (0.58-1.17)	0.8 (0.56-1.12)	0.93 (0.65-1.30)	1.29 (0.99-1.66)
Other	1.31 (0.93-1.82)	1.21 (0.86-1.66)	0.9 (0.60-1.28)	0.92 (0.62-1.32)	1.26 (0.96-1.63)
**Active military duty**	1.06 (0.74-1.53)	1.09 (0.76-1.59)	1.11 (0.77-1.62)	**1.75 (1.15-2.74)**	**1.50 (1.10-2.07)**
**Age**	0.99 (0.98-1.00)	1.00 (0.99-1.01)	1.00 (0.99-1.02)	1.01 (1.00-1.03)	1.00 (0.99-1.02)
**BMI**	**1.03 (1.01-1.05)**	1.00 (0.98-1.03)	1.01 (0.98-1.03)	1.01 (0.98-1.04)	1.00 (0.98-1.02)
**Depression (PHQ-9 continuous scores)**		**1.20 (1.18-1.22)**	**1.21 (1.17-1.24)**	**1.15 (1.12-1.19)**	**1.09 (1.07-1.12)**
**Anxiety (GAD-7 continuous scores)**	**1.2 (1.18-1.22)**		0.97 (0.94-1.00)	**1.05 (1.02-1.09)**	**1.05 (1.02-1.08)**

***p < 0.001; **p < 0.01; *p < 0.05

[1] aRR- adjusted rate ratio; CI- confidence interval

[2] All models adjusted for time since symptom onset or enrollment, sex, race/ethnicity, active duty military, age, BMI, disease severity, vaccine breakthrough, and concurrent depression and anxiety scores from PHQ-9 and GAD-7 questionnaires

[3] Statistically significant aRR at p<0.05 are in bold text

[4] PHQ-9 has a cutoff score of ≥10 to identify participants with moderate-to-severe depression

[5] GAD-7 has a cutoff score of ≥10 to identify participants with moderate-to-severe anxiety

[6] PROMIS® 8a Fatigue questionnaire has a cutoff of ≥60 to identify participants one standard deviation above the sample mean

[7] PROMIS® cognitive function short form 4a has a cutoff of ≤40 to identify participants one standard deviation below the sample mean

[8] PROMIS® cognitive function short form abilities subset 4a has a cutoff of ≤36 to identify participants one standard deviation below the sample mean
