# Supplementary material for: SARS-CoV-2 infection is associated with self-reported post-acute neuropsychological symptoms within six months of follow-up
Source: PLoS One. 2024 Apr 16;19(4):e0297481. doi: 10.1371/journal.pone.0297481 (PMC11020833; doi:10.1371/journal.pone.0297481)
Supplement: S2 Table — (PDF) [file pone.0297481.s002.pdf]

S2 Table. Depression, anxiety, fatigue, self-assessed cognitive function, and demographics of EPICC study participants by history of anxiety and/or depression diagnosis

	Total (N=2383)	Prior diagnosis of depression/anxiety (N=863)	None (N=1496)	P-value[1]
**Outcomes, n (%)**				
Depression (PHQ-9 score ≥ 10)	281 (11.8%)	197 (23.7%)	84 (5.4%)	< 0.001
Anxiety (GAD-7 score ≥ 10)	279 (11.7%)	207 (24.9%)	72 (4.6%)	< 0.001
Fatigue (PROMIS® 8a t-score ≥ 60)	274 (11.5%)	163 (19.6%)	111 (7.2%)	< 0.001
Poor cognitive function (PROMIS® 4a t-score ≤ 40.29)	254 (10.7%)	168 (20.2%)	86 (5.5%)	< 0.001
Poor cognitive function abilities (PROMIS® subset 4a t-score ≤ 36.07)	444 (18.6%)	232 (27.9%)	212 (13.7%)	< 0.001
**SARS-CoV-2 Positive**	687 (28.8%)	264 (31.8%)	423 (27.3%)	0.020
**Male, n (%)**	1558 (65.4%)	451 (54.3%)	1107 (71.3%)	< 0.001
**Race/ethnicity, n (%)**				0.002
White	1564 (65.6%)	526 (63.3%)	1038 (66.9%)	
Black	159 (6.7%)	75 (9.0%)	84 (5.4%)	
Hispanic or Latino	318 (13.3%)	121 (14.6%)	197 (12.7%)	
Other	342 (14.4%)	109 (13.1%)	233 (15.0%)	
**Active military duty, n (%)**	1958 (82.2%)	664 (79.9%)	1294 (83.4%)	0.035
**Age, mean (SD)**	37.5 (10.3)	37.0 (9.6)	37.7 (10.7)	0.323
**BMI, mean (SD)**	27.7 (4.6)	28.5 (5.0)	27.2 (4.3)	< 0.001
**Months post symptom onset, mean (SD)**	2.4 (1.9)	2.5 (1.9)	2.4 (1.9)	0.068

[1]Comparing participants with and without a prior diagnosis of depression and/or anxiety using chi-square tests
